# Cannabinoid exposure during zebra finch sensorimotor vocal learning persistently alters expression of endocannabinoid signaling elements and acute agonist responsiveness

**DOI:** 10.1186/1471-2202-12-3

**Published:** 2011-01-06

**Authors:** Ken Soderstrom, Justin L Poklis, Aron H Lichtman

**Affiliations:** 1 Department of Pharmacology and Toxicology, Brody School of Medicine, East Carolina University, Greenville, NC 27834, USA; 2 Department of Pharmacology and Toxicology, Virginia Commonwealth University, Richmond, Virginia 23298, USA

## Abstract

**Background:**

Previously we have found that cannabinoid treatment of zebra finches during sensorimotor stages of vocal development alters song patterns produced in adulthood. Such persistently altered behavior must be attributable to changes in physiological substrates responsible for song. We are currently working to identify the nature of such physiological changes, and to understand how they contribute to altered vocal learning. One possibility is that developmental agonist exposure results in altered expression of elements of endocannabinoid signaling systems. To test this hypothesis we have studied effects of the potent cannabinoid receptor agonist WIN55212-2 (WIN) on endocannabinoid levels and densities of CB_1 _immunostaining in zebra finch brain.

**Results:**

We found that late postnatal WIN treatment caused a long-term global disregulation of both levels of the endocannabinoid, 2-arachidonyl glycerol (2-AG) and densities of CB_1 _immunostaining across brain regions, while repeated cannabinoid treatment in adults produced few long-term changes in the endogenous cannabinoid system.

**Conclusions:**

Our findings indicate that the zebra finch endocannabinoid system is particularly sensitive to exogenous agonist exposure during the critical period of song learning and provide insight into susceptible brain areas.

## Background

Zebra finches learn a song during distinct periods of vocal development [1]. Exposure to cannabinoid agonists during these periods alters vocal development by reducing both song stereotypy and the number of notes incorporated into mature song produced in adulthood [2,3]. This, combined with evidence for distinct developmental regulation of CB_1 _cannabinoid receptor expression during periods of song learning [4], suggests a role for endogenous cannabinoid signaling in normal vocal developmental processes. Vocal learning and production in zebra finches is associated with marked physiological changes within distinct regions of telencephalon (e.g. lMAN, Area X, auditory Field L2, RA) and thalamus (DLM, ovoidalis) known to be critical for song perception, production and learning. Each of these regions distinctly and densely expresses CB_1 _receptors [3]. Normal development in song regions is associated with gross anatomical changes in region volume, neuron number and density, both increases and decreases in axonal interconnections between song regions, and changes in synaptic densities. Cannabinoid-altered vocal development implies that exogenous agonist exposure must somehow alter some or all of these processes responsible for critical periods of song learning. We are currently working to identify which processes are modified by developmental cannabinoid exposure and the mechanism(s) responsible.

Distinct song region CB_1 _receptor expression implies a role for endocannabinoid signaling in vocal learning. The endocannabinoid system is a *bona fide *neurochemical signaling system comprising at least two G-protein-coupled receptors (CB_1 _and CB_2_, with the former expressed at much higher density in CNS), and an array of fatty acid ligands, most notably anandamide and 2-arachidonyl glycerol (2-AG), that are capable of activating the receptors. Like most cell signaling systems, cannabinoid receptors and endocannabinoid ligands are subject to biochemical regulation of synthesis, expression and metabolic breakdown [5]. A particularly notable form of G-protein-coupled receptor regulation is agonist-induced internalization and metabolism, whereby the duration of agonist effects are limited by decreased receptor expression [6]. Thus, a potential mechanism for exogenous cannabinoid-induced alteration of normal development may involve endocannabinoid and receptor regulation. This hypothesis was tested through the experiments described below.

## Results

### CB_1 _immunostaining

**Figure 1:**
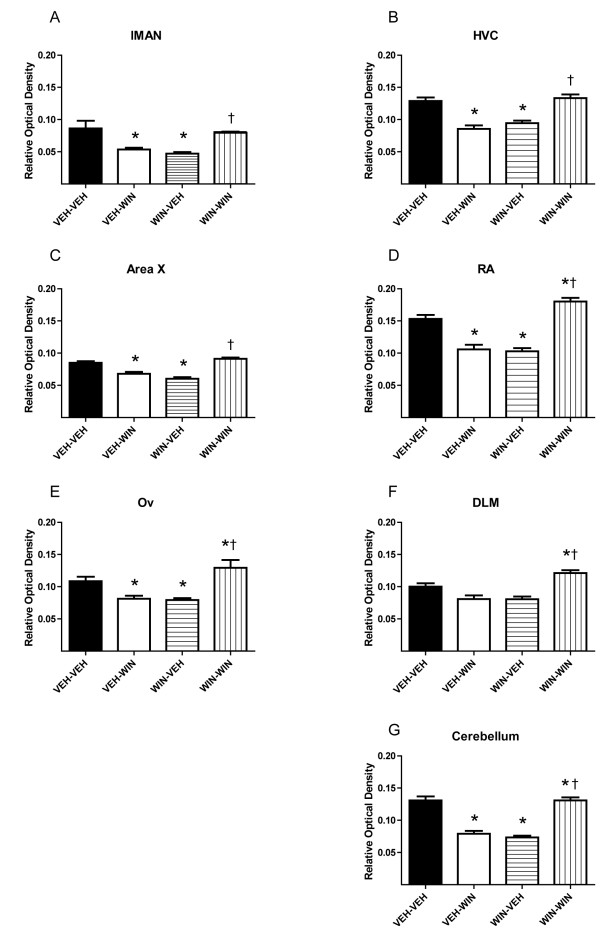
**Developmental cannabinoid exposure alters CB_1 _immunostaining within various zebra finch brain regions**. Initial daily treatments over 25 days are indicated by first designations (VEH-, WIN-). Later, single acute treatments in adulthood are indicated second (-VEH, -WIN, see Table 1). Treatments were delivered during sensorimotor song learning (from 50-75 days) and measured in adulthood (> 100 days). Basal levels of staining are decreased following repeated WIN exposure during development in all regions but DLM (compare VEH-VEH to WIN-VEH). Acute responsiveness is increased in all regions (compare VEH-WIN to WIN-WIN). Asterisks indicate differences from VEH-VEH treatment groups (*p *< 0.05, one-way ANOVA followed by SNK post-tests). Daggers indicate differences from VEH-WIN groups, double-daggers indicate differences from WIN-VEH.

**Figure 2:**
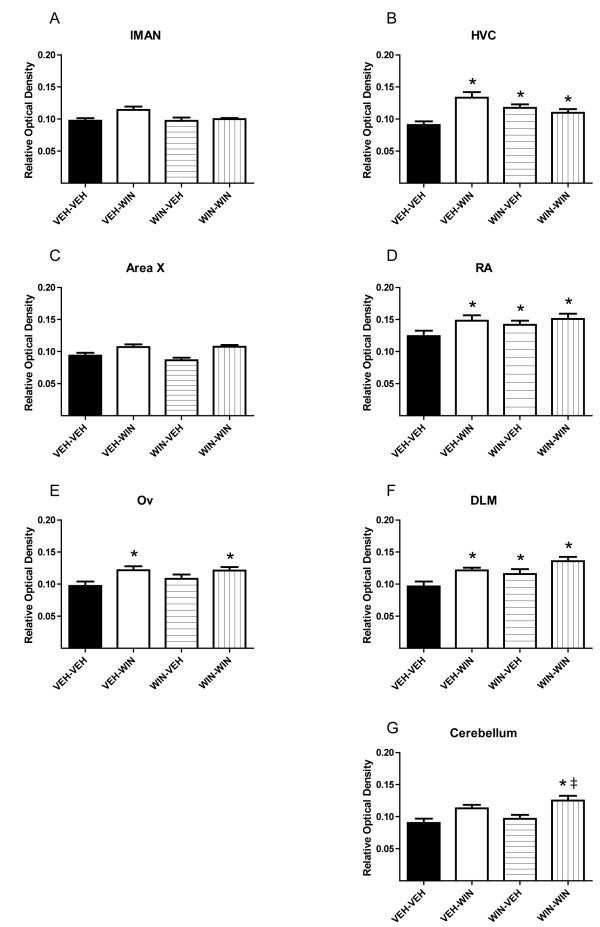
**Repeated cannabinoid treatment during adulthood alters CB_1 _immunostaining primarily within vocal-motor-related regions of zebra finch brain (HVC, RA, and DLM). **Initial daily treatments over 25 days are indicated by first designations (VEH-, WIN-). Later, single acute treatments are indicated second (-VEH, -WIN, see Table 1). Basal staining levels are increased following repeated WIN exposure in adulthood in (B) HVC, (D) RA, and (F) DLM (compare VEH-VEH to WIN-VEH in these panels). Acute responsiveness is not modified following repeated treatments (compare VEH-WIN and WIN-WIN). Chronic treatment did increase responsiveness within the molecular layer of the cerebellum (double-dagger, panel G) Asterisks indicate differences from VEH-VEH treatment groups (*p *< 0.05, one-way ANOVA followed by SNK post-tests).

Relative optical densities of CB_1 _immunostaining within selected brain regions are summarized by treatment groups in Figures 1 and 2. Persistent effects of chronic, 25-day WIN treatments on CB_1 _staining densities were assessed by comparing VEH-VEH and WIN-VEH groups. In the case of animals treated during sensorimotor development (Figure 1), repeated daily WIN treatments led to a significant decrease in staining density within all regions except DLM (differences from VEH-VEH group indicated by an asterisk). This contrasts with chronic treatment effects produced in adult animals where chronic WIN treatments either produced no effect (lMAN, Area X, Ov, cerebellum) or increased staining densities (HVC, RA, DLM, see Figure 2).

Acute effects of WIN treatments (given 90 min prior to perfusion) on staining densities were assessed by comparing VEH-VEH and VEH-WIN groups. Following developmental vehicle injections and maturation to early adulthood, acute WIN treatments decreased CB_1 _immunostaining densities in all brain regions except DLM. A different pattern of acute responsiveness was observed in animals treated in adulthood: acute increases in staining densities were observed within HVC, RA, DLM and Ov. Acute responsiveness following repeated WIN administration was assessed by comparing WIN-VEH to WIN-WIN groups. In the case of animals treated with WIN during development, acute WIN exposure resulted in significantly increased CB_1 _immunostaining densities in all brain regions studied (Figure 1, differences indicated by a double-dagger). This effect of acute WIN to increase staining densities following chronic WIN differed with responses observed following chronic VEH in every brain region (Figure 1, compare VEH-WIN to WIN-WIN, differences indicated by a dagger). Following repeated WIN treatments of adults, no acute WIN effect was detected in any region except within the molecular layer of cerebellum where densities were increased following earlier chronic WIN exposure (Figure 2G, compare WIN-VEH to WIN-WIN, difference indicated by a double-dagger).

### Effects on brain 2-AG levels

**Figure 3:**
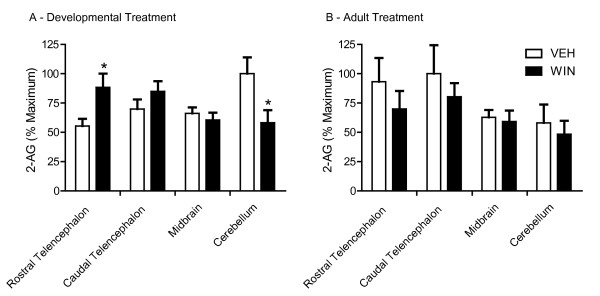
**Effects of chronic, developmental, cannabinoid treatments on endogenous 2-AG levels in zebra finch brain. **Brains were rapidly dissected into rostral (Rostral Tel.) and caudal (Caudal Tel.) telencephalon, midbrain and cerebellum. Lipids were extracted, spiked with deuterium-labeled internal standards and subjected to LC-ESI-MS-MS for quantitative analysis of 2-arachidonyl glycerol (2-AG) content. (A): ANOVA indicates a significant relationship between brain region and 2-AG content (*p *< 0.05). Post-hoc analysis reveals a significant increase in 2-AG content in rostral telencephalon (which contains the song regions lMAN and Area X). Significant decreases in endocannabinoid content within cerebellum were observed following developmental WIN (*p < 0.05). (B): No effect of repeated WIN treatments given during adulthood on 2-AG levels were found.

Effects of repeated WIN treatments on levels of the endocannabinoid 2-AG in various brain regions were assessed by LC-ESI-MS-MS. Results are summarized in Figure 3. WIN exposure during sensorimotor development significantly increased 2-AG levels in rostral telencephalon (containing the song regions lMAN and Area X, Figure 3A). Levels were unchanged in caudal telencephalon (containing vocal motor regions, HVC and RA) and thalamus/midbrain (containing Ov and DLM). In contrast to effects produced in rostral telencephalon, developmental WIN exposure decreased 2-AG content within cerebellum. No effects of repeated WIN treatments on 2-AG levels in adult animals were observed in any of the brain regions studied (Figure 3B).

## Discussion

Results reported herein demonstrate that developmental cannabinoid exposure persistently disregulates endocannabinoid signaling at both the receptor and endogenous agonist levels. This disregulation is significant because it's accompanied by altered vocal development that is produced by the same cannabinoid treatments [2,7]. These findings are among the first to associate cannabinoid-altered behavioral development with persistently-altered neurophysiology.

More work in evaluating persistent cannabinoid effects has employed cell and tissue culture systems. For example, in *ex vivo *cultures of rat hippocampi, periods of 24 hr WIN exposure dose-dependently reduces CB_1 _immunostaining [8] which is consistent with persistent effects of chronic WIN that we find after developmental, but not adult exposure (compare VEH-VEH and WIN-VEH groups, Figures 1 and 2). It may be important that most *ex vivo *tissue culture systems employ tissue obtained from immature animals. For example, in the case cited above, hippocampi were isolated from rat pups at post-natal day two. Therefore, *ex vivo *cultures of CNS tissue may more closely model effects on the developing CNS than the mature, adult system. Interestingly, rat and zebra finch development occur over similar periods, with 'adolescence' reached between 35 - 45 days, and early adulthood by about 90 days [7,9].

In cultures of cells heterologously expressing CB_1 _receptors, it has been appreciated for some time that agonist stimulation promotes a rapid internalization of receptor protein [10]. In our histological model, tissue is permeabilized, allowing antibody access to both plasma membrane-delimited and potential intracellular pools of receptor protein, making it unlikely that the rapid (90 min post-treatment) changes in staining density that we observe are due to acute cellular translocation of receptor protein (although this may very well be occurring). A more likely mechanism for rapid changes in antibody staining may involve changes in epitope access related to interaction with other cellular proteins. The peptide used to produce our anti-zebra finch CB_1 _antibody represents the first 16 amino acids of the intracellular tail region of the receptor [3], a domain implicated in signal transduction and interaction with intracellular regulatory proteins [11]. Fixation and cross-linking of tissue with receptors coupled to such regulatory or signal transduction proteins may occlude epitope access, reducing antibody interaction and resulting in reduced staining intensity. Thus, differences in staining densities that we have documented may be more indicative of differential effects on CB_1 _interaction with other proteins than on receptor abundance itself. Of course we cannot exclude the possibility that decreased immunostaining may indicate receptor degradation and/or reduced expression, as both phenomenon are known to occur following agonist activation [10].

It is interesting that acute WIN treatment of birds chronically treated with vehicle control injections responded differently following late-postnatal and adult treatments (for example, see VEH-WIN responses in Figures 1B and 2B). We observed similar differential effects of control treatments on acute changes in FoxP2 expression [12]. These differences may be attributable to distinct sensitivity to handling stress during sensorimotor development, but not adulthood. Alternatively, a differential sensitivity to visual isolation in developing vs. adult animals may have contributed. Following developmental treatment, birds were housed in visual isolation to prevent potential song learning from other subjects (animals treated as adults were also isolated for the same period as part of the control treatment). This isolation lasted 25 days, encompassing late-sensorimotor development to early adulthood (75-100 days). Zebra finches are social, flocking birds, and so isolation during development may have produced stress [13]. The potential for handling- and isolation-induced effects on vocal development is a question worthy of additional study. It is notable that accumulating evidence supports a role for endocannabinoid signaling in stress responses and fear extinction [14].

**Figure 4:**
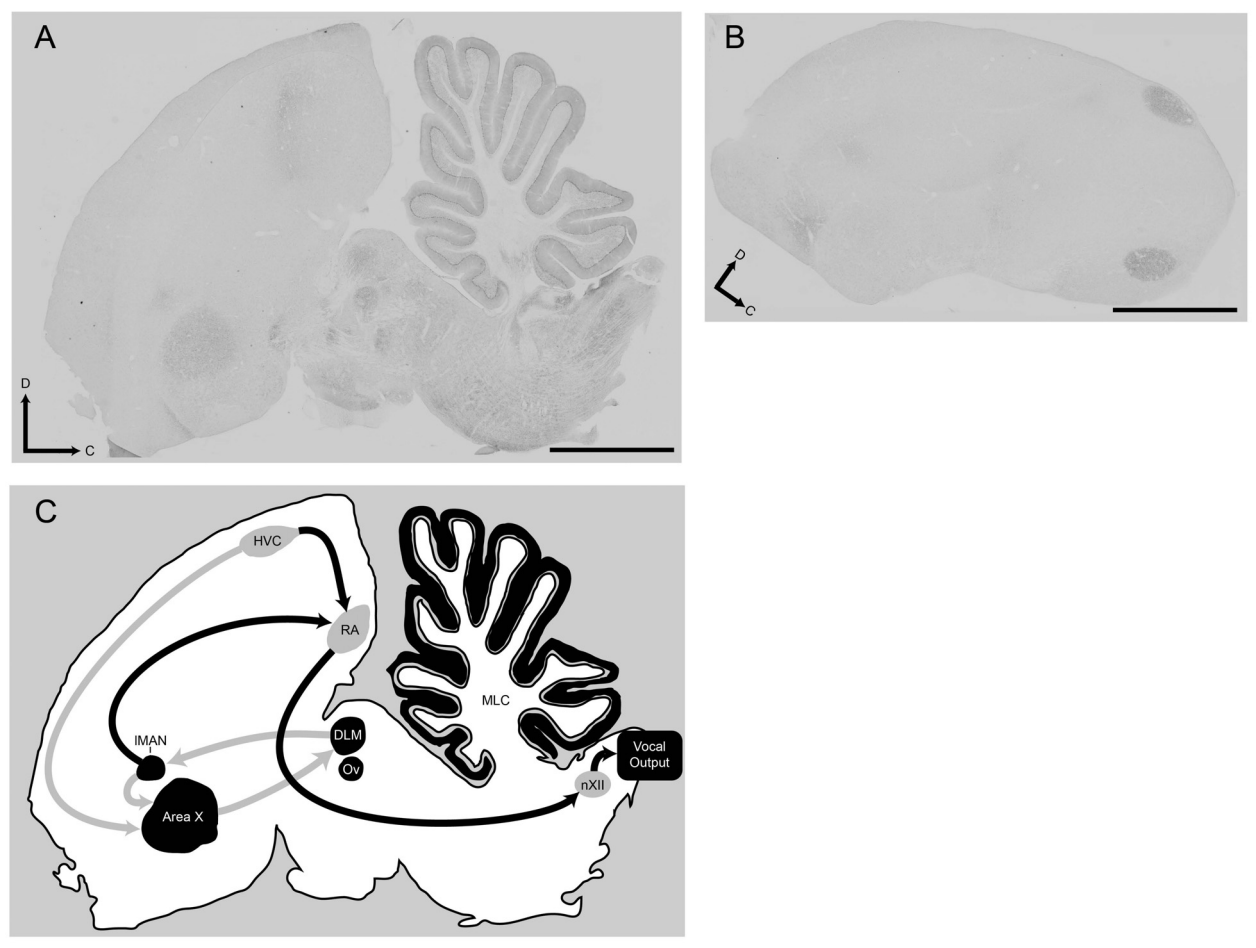
**Representative CB1 immunostaining. **(A) Medial parasagittal sections contain rostral song regions lMAN and Area X, thalamic regions DLM and Ov, and cerebellum. (B) More lateral parasagittal sections capture HVC and RA. Dorsal and caudal are indicated by arrows, the bars = 1 mm. (C) A tracing of the micrograph in panel A serves as a diagram summarizing relative locations of song regions studied. Regions present in panel A are represented in black, other regions are diagrammatically represented in grey (HVC, RA and nXII). Established interconnections between song regions are indicated with arrows. Grey arrows indicate rostral forebrain circuitry essential for vocal learning. Black arrows indicate caudal vocal-motor circuitry.

Despite the confounding effects of control treatments, chronic effects of repeated WIN exposure on later acute WIN sensitivity are clear. Following developmental WIN treatment, staining densities were reduced in all of the brain regions except DLM (Figure 1, compare VEH-VEH to WIN-VEH). Following adult treatments, significant increases were seen in only three regions: HVC, RA, and DLM. HVC and RA are vocal motor regions of caudal telencephalon (see Figure 4). Activity in HVC drives RA which outputs to the nucleus of the twelfth cranial nerve which innervates the syrinx, eliciting vocalization [15]. The thalamic region, DLM, modulates this caudal vocal-motor output indirectly through projection to the rostral song region, lMAN, that in-turn, projects to RA [16]. Distinct cannabinoid effects in these vocal-motor regions during adulthood further suggests a normative role for cannabinoid signaling in song production, a conclusion reinforced by earlier evidence that acute WIN decreases vocal output in adults [17]. In addition to modulating vocal output in adulthood, the rostral song regions lMAN and Area X are essential for vocal learning during development [18]. More generalized effects within all song regions but DLM following developmental cannabinoid exposure, underscores the distinct sensitivity to cannabinoid agonism during periods of zebra finch vocal learning.

On the surface it appears that developmental exposure-related increased levels of the endocannabinoid, 2-AG, and decreased CB_1 _immunostaining are contradictory (compare Figure 3A and Figure 1A and 1C). However, if as proposed above, staining is more a function of receptor state than abundance, it follows that persistently decreased staining following developmental agonist exposure may be due to interaction with signal transduction proteins that antagonize binding of the antibody. Such coupling may actually increase the population of functional CB_1 _receptors. If this is the case, then acute increases in staining following WIN challenge of animals developmentally exposed may indicate uncoupling from other cellular proteins and decreased responsiveness (compare WIN-VEH and WIN-WIN groups in all panels of Figure 1, note that similar increases were not seen following adult treatment, Figure 2). Assays of CB_1 _receptor function following developmental treatments will be required to clearly test this hypothesis [19].

Reports of rapid changes in expression of endocannabinoid signaling elements *in vivo *have not been widely reported, and this is a key contribution of the work reported herein. Most prior reports of altered expression of cannabinoid signaling elements *in vivo *involve studies of regulation of feeding behavior. In zebra finches, we have found that brief periods of food deprivation are associated with increased brain levels of 2-AG [3]. In pancreatic islet cells of Wistar rats, overnight periods of food deprivation are associated with increased expression of CB_1 _receptors, an effect rapidly reversed by oral glucose [20].

Unlike food restriction-induced general increases of 2-AG levels in all regions of brain except cerebellum [3], developmental WIN exposure increased 2-AG selectively within rostral telencephalon and decreased levels in cerebellum (Figure 3A). These results indicate that cannabinoid-altered vocal development is associated with persistent enhancement of endocannabinoid signaling within rostral telencephalon. This is interesting as the song regions of rostral telencephalon (lMAN and Area X, see Figure 4), are not essential for production of learned song [21,22], and seem to perform a modulatory function related to song timing, that perhaps serves as an error-correcting mechanism [16]. Decreased 2-AG content within cerebellum is notable, as this region has not typically been associated with vocal development. Potential persistent effects of developmental cannabinoid exposure on motor function warrants further investigation, and recent evidence suggests that cerebellum is more important to sensory integration and cognition in zebra finches than previously thought [23]. In mice, functional changes in cerebellar sensitivity to cannabinoid drugs have been observed as a function of tolerance and following antagonist-precipitated withdrawal [24]. This raises the possibility that similar effects of chronic treatment are produced following cannabinoid-altered vocal learning, and may contribute to altered developmental course.

Production of opposing effects on 2-AG levels across CNS regions by the same drug is interesting, and may be attributable to differing neurochemistry within each area (e.g. rostral telencephalon vs. cerebellum). A growing consensus supports a presynaptic inhibitory role for endocannabinoid activation of CB_1 _to reduce the probability of neurotransmitter release. Because presynaptic CB_1 _activation inhibits release of both excitatory (e.g. glutamatergic) and inhibitory (e.g. GABAergic) transmitters, regional cannabinoid effects may depend upon relative levels of excitatory vs. inhibitory tone (reviewed by [25]). For example, across all species studied, CB_1 _densities are particularly high within the molecular layer of cerebellum [26]. This region is largely comprised of the axonal parallel fibers that rise from cerebellar granule cells. These parallel fibers synapse on Purkinje cell dendritic arbors, primarily releasing excitatory glutamate (reviewed by [27]). Presynaptic CB_1 _activation produced by repeated WIN treatments would be expected to reduce the probability of excitatory glutamate release, effectively reducing neural activity, and decreasing post-synaptic release of 2-AG. In the case of rostral telencephalon, distinct and dense CB_1 _receptors are expressed within the prominent Area X of striatum [7]. CB_1 _expression is particularly dense within Area X at 50-75 days of age, the period during which exogenous WIN treatments were administered in our current studies [28]. In contrast to molecular layer of cerebellum, Area X is characterized by significant inhibitory GABAergic transmission [29,30]. Thus, cannabinoid agonism may be expected to reduce inhibitory signaling within Area X, leading to increased neuronal activity and post-synaptic 2-AG release.

## Conclusions

In summary, repeated cannabinoid agonist exposure during zebra finch sensorimotor vocal development was associated with increased levels of the endocannabinoid, 2-AG, and decreased CB_1 _immunostaining intensities within regions of rostral telencephalon. Similar changes were not observed following repeated WIN treatment of adult animals, demonstrating distinct developmental cannabinoid sensitivity during vocal development. Developmental WIN exposure also altered acute responsiveness to WIN challenge, an effect that was not seen following chronic treatment of adults. These findings suggest that the cannabinoid-altered vocal development involves persistent changes in expression, regulation and responsiveness of endocannabinoid signaling elements. In the context of the developing zebra finch CNS, these endocannabinoid signalling elements are particularly susceptible to disregulation caused by prolonged exposure to cannabinoids.

## Methods

### Animals

Male zebra finches bred in our aviary and sexed at ~ 25 days via PCR [31] were used in these experiments. Except where indicated otherwise, six animals were assigned to each treatment group. Prior to the start of experiments, birds were housed with an adult male song tutor in flight aviaries and provided free access to mixed seeds (SunSeed VitaFinch), grit, water, and cuttlebone. Each flight aviary contained several perches. The light-dark cycle was controlled at L:D 14:10 h and ambient temperature was maintained at 78°F.

Animals were cared for and experiments conducted according to protocols approved by East Carolina University's Animal Care and Use Committee.

### Treatments

Drug treatments were given by intramuscular injection of 50 μl into the pectoralis muscle. Drug dilutions for injection were made from 10 mM stocks (in DMSO) to produce a final vehicle of 1:1:18 DMSO:Alkamuls (Rhodia, Cranberry, NJ):PBS (pH = 7.4). Because zebra finches are inactive and don't sing in the dark, treatments were given immediately prior to the beginning of light cycles to avoid potential song- and activity-related changes in receptor expression. Prior work investigating immediate early gene expression experiments indicated that peak agonist-induced immunoreactivity of both ZENK and FoxP2 occurs 90 min following treatment with the potent CB_1_/CB_2 _receptor antagonist WIN55,212-2 (WIN) and therefore this period was adopted to investigate potential acute changes in CB_1 _immunostaining [32,33].

For developmental experiments, once-daily injections of vehicle (VEH- groups) or 1 mg/kg WIN (WIN- groups) were given to male zebra finches from 50 to 75 days of age (during the sensorimotor period of zebra finch vocal learning). WIN treatment during this period is well-documented to alter both song stereotypy and incorporation of notes into mature song (see [34,35]). Following completion of treatments, animals were allowed to mature to at least 100 days of age in visual isolation. Upon maturation, groups of animals were either given a single acute vehicle injection (VEH-VEH and WIN-VEH groups), or given a single acute injection of 3 mg/kg WIN in order to induce potential changes in CB_1 _immunoreactivity (VEH-WIN and WIN-WIN). Groups of adults (n = 4) were treated similarly to control for effects dependent on developmental exposure. Note that treatment groups are designated by the repeated, developmental treatment indicated first, and separated by a hyphen from the indication of a second final acute treatment given in adulthood. Treatment groups and designations are summarized for clarity in Table 1.

**Table 1:** Summary of Treatment Groups

		Chronic Tx	Acute Tx
Abbreviation	# Animals	(QD for 25 days)	1× in adulthood
VEH-VEH	6 LPN*, 4 Adult	Vehicle	Vehicle
VEH-WIN	6 LPN*, 4 Adult	Vehicle	3 mg/kg WIN
WIN-VEH	6 LPN*, 4 Adult	1 mg/kg WIN	Vehicle
WIN-WIN	6 LPN*, 4 Adult	1 mg/kg WIN	3 mg/kg WIN

*LPN, Late-Postnatal Treatment

### CB_1 _immunostaining

Ninety minutes following acute treatments, birds were killed by Equithesin overdose and transcardially perfused with phosphate-buffered saline (PBS, pH = 7.4) followed by phosphate-buffered 4% paraformaldehyde, pH = 7.0. After brains were removed and immersed overnight in buffered 4% paraformaldehyde, they were blocked down the midline and left hemispheres were sectioned parasagittally (lateral to medial) on a vibrating microtome. Immunohistochemistry was performed using a standard protocol reported in [36]. For immunohistochemistry experiments, 30 micron sections of zebra finch brain were reacted with a 1:3000 dilution of polyclonal anti-zebra finch CB_1 _antibody raised in rabbit. The selectivity of this antibody for zebra finch CB_1 _has been demonstrated previously, and it has been used in multiple studies [3,4,37]. Tissue sections were rinsed in 0.1% H_2_O_2 _for 30 min, blocked with 5% goat serum for 30 min, and reacted overnight in blocking solution containing anti-zebra finch CB_1 _antibody (1:3000). After antibody exposure, sections were rinsed in PBS (pH = 7.4), incubated in blocking solution containing biotinylated goat anti-rabbit antiserum (1:500) for 1 hour, rinsed with PBS again, and then submerged in avidin-biotin-peroxidase complex solution (purchased as a kit from Vector Laboratories, cat # PK-4005) for 1 hour. Antibody labelling was visualized with DAB solution (Vector cat # SK-4100). Control sections that were not reacted in primary antibody were not immunoreactive. To eliminate possible variance associated with reaction conditions, tissue from equal numbers of animals from each treatment group within an experiment were processed simultaneously.

Staining was examined in various brain regions at 40 × using an Olympus BX51 microscope under brightfield conditions. Multiple images were captured using a Spot Insight QE digital camera and Image-Pro Plus software (MediaCybernetics, Silver Spring, MD) under identical, calibrated exposure conditions. These images were background-corrected and converted to grey scale (see Figure 4). The borders of brain regions were traced manually. In cerebellum, only molecular layer staining was measured. Mean optical densities of brain areas enclosed within traced regions were determined without knowledge of treatment condition from five separate sections per animal using Image-Pro Plus software. Measurements were made independently by two investigators and pooled for analysis. Mean optical densities within each region were compared across treatment group using one-way ANOVA as described below.

### Determination of 2-arachidonylglycerol (2-AG) content

Groups of male zebra finches were randomly assigned to receive vehicle or 1 mg/kg WIN injections from 50 to 75 days of age (n = 4 animals per treatment). Following these developmental treatments, birds were allowed to mature to adulthood (100 days of age) in visual, but not auditory isolation. To assess dependence of effects on developmental exposure a separate experiment was done with adult animals (n = 4) employing a similar 25-day treatment period followed by visual isolation. Following treatment and maturation, birds were killed by anesthetic overdose and brains rapidly removed to ice. Brains were blocked down the midline, and tissue from each hemisphere was extracted and analyzed independently resulting in two data points per sample per bird. Developmental experiments were performed in triplicate resulting in a final n = 24 per treatment. Tissue from each brain half was rapidly dissected into: rostral (containing the song regions lMAN [lateral magnocellular nucleus of anterior nidopallium] and Area X of striatum); and caudal (containing the song regions HVC and RA [robust nucleus of arcopallium] and auditory regions L2 and NCM) telencephalon; cerebellum; and midbrain and immediately frozen in liquid nitrogen. Frozen tissue was stored at -80°C until extracted as described previously (Matias et al., 2003). Briefly, tissues were homogenized in and extracted with chloroform:methanol:Tris-HCl 50 mM, pH = 7.5, 2:1:1, (v/v) containing internal standards (10 pmol of anandamide-d8, palmitoylethanolamine-d4 and 100 pmol of 2-arachidonyl glycerol-d5, obtained from Cayman Chemicals). The lipid-containing organic phase was collected and dried. Samples were reconstituted in 100 μL of 10:90 (v/v) water: methanol with 0.1% ammonium acetate and placed in autosample vials for analysis.

The electrospray ionization-mass spectrometry-mass spectrometry (LC-ESI-MS-MS) method used to detect and quantitative anandamide and 2-arachidonyl glycerol (2-AG) was a modification of a previously published method [38]. The LC-ESI-MS-MS system used was Shimadzu Shumadzu Prominence LC system coupled to an Applied Bio Systems 3200 Q trap with a turbo V source for TurbolonSpray. The MS analyses were carried out in the multiple reaction monitoring mode and the following transition ions were monitored: (348 > 62) and (348 > 91) for anandamide; (356 > 62) for Anandamide -d8; (379 > 287) and (279 > 269) for 2-AG; (384 > 96) for 2-AG-d5. The analytical column used was a Discovery ^® ^HS C18, 4.6 mm × 15 cm, 3 micron (Supelco, USA). The mobile phase consisted of (10:90) water: methanol with 0.1% ammonium acetate and 0.1% formic acid and a flow rate of 0.3 mL/min was used. A negative control and seven point calibration curves at concentrations of 0, 0.25, 0.5, 1, 2, 4, 8 pmoles for 2-AG and 0, 0.038, 0.075, 0.15, 0.3, 0.6, and 1.2 pmoles for anandamide were prepared with each analytical run. Sample concentrations were calculated by linear regression.

### Statistical Analyses

Because sections containing all brain regions were reacted together, and each antibody reaction contained tissue from all four treatment groups, relationships between drug treatments and within-region CB_1 _immunostaining optical densities were determined through two-way ANOVA by treatment and brain region. Following ANOVA determination that mean relative optical density values differed across treatment, Student-Newman-Keuls post-tests were done to determine brain regions with that differed by treatment. In the case of 2-AG measurements, brain areas were processed independently, and therefore mean content of each area by vehicle or WIN treatment groups were compared using t-tests.

## Authors' contributions

KS was responsible for designing and conducting the immunohistochemical experiments presented. AHL and JLP contributed to the design of 2-AG assay experiments, which were conducted by JLP. All authors have read and approve the final manuscript.
